# Integrating Equity into Health Information Systems: A Human Rights Approach to Health and Information

**DOI:** 10.1371/journal.pmed.0020102

**Published:** 2005-04-26

**Authors:** Lexi Bambas

## Abstract

Health information systems can play a crucial role in supporting human rights by documenting and tracking health and health inequities, and by creating a platform for action and accountability

One of the most fundamental human rights is the assumption that each person matters, and everyone deserves to be treated with dignity—this is the tenet from which all other human rights flow. Another is that those who are most vulnerable deserve special protection. However, in many developing countries, vast numbers of children are born but never counted, and their health and welfare throughout their lives remains unknown. And because single-mean measures of population health mask inequalities among the best-off and worst-off, the health of vulnerable populations is not effectively documented and acknowledged. Health information systems can play an important role in supporting these rights by documenting and tracking health and health inequities, and by creating a platform for action and accountability.

A human rights approach to health information systems also supports effective health development. To effectively improve population health, governments and communities need access to socioeconomically disaggregated population health data. Because the relationship between such information and human rights has received little attention, the two areas of health information systems and human rights have done little to support each other.

## The Health Metrics Network

The Health Metrics Network (HMN) is a global collaboration focused on strengthening country health information systems to generate sound data for decision-making at country and global levels (see http://www.who.int/healthmetrics/en/). Its interim secretariat is based at the World Health Organisation. The development group of HMN recently considered strategies for strengthening health information systems within countries. The Equity Working Group of HMN made recommendations that outlined the content of equity-sensitive information systems, identified opportunities for minimizing collection burdens, and suggested strategies to foster an equity-oriented decision-making culture. Although the recommendations were implicitly focused on human rights and on improving opportunity for the worst-off populations, making that framework explicit helps us to acknowledge and clarify the values on which decisions for developing information systems are made. The framework is based on several principles rooted in human rights and their implied actions (see Sidebar).

**Principles to Guide a Human Rights-Oriented Framework for Integrating Equity into Health Information Systems**

1. **Each person has dignity and each matters**
  —Count everyone in society from birth to death
2. **Everyone should have opportunities for health *and the means to improve health*; vulnerable populations deserve special attention**
  —Collect and analyze information related to inequalities in health status and determinants of health among various better-off and worse-off subpopulations
3. **Governments are accountable to the public, communities have a right to the information they need to make healthy decisions, and individual autonomy should be supported**
  —Release information to the public in a meaningful form
4. **Governments, communities, and individuals are all responsible for promoting health and health opportunities**
  —Support capacity for and cultures of human rights-oriented decision-making based on health information

## Every Individual Matters

**Figure 1:**
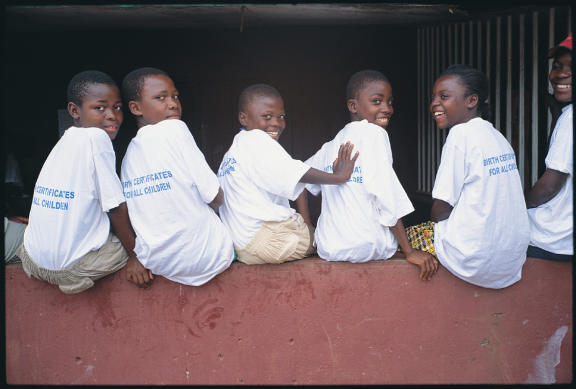
Every Birth Must Be Registered This photo comes from the development organization Plan (www.plan-international.org), which has launched an international campaign for universal birth registration (www.writemedown.com). The campaign's Web site states that a third of babies born each year are not registered (this statistic comes from Unicef), and that many of these unregistered children have no legal right to health care, education, or the state's protection. (Photo: Adam Hinton/Plan)

Despite the acknowledged importance of counting everyone, only 57 of the 192 WHO member states, almost all of which are developed countries, have vital registration systems that report on births and deaths for at least 90% of the population. Consequently, a primary recommendation from the Equity Working Group is that health information systems should support the most basic acknowledgment of human rights—one's existence—by counting births and deaths in every country through a vital registration system (Figure 1).

## Opportunities and the Means to Health

A person's human rights are recognized to include “a standard of living adequate for the health and well-being of himself and of his family, including food, clothing, housing, and medical care and necessary social services, and the right to security in the event of…lack of livelihood in circumstances beyond his control” [1]. In other words, the means necessary to achieve health are part and parcel of a right to health. However, many country studies have repeatedly shown that there are large gaps between the opportunities of advantaged and disadvantaged sub-populations. For example, in South Africa, average household expenditure for whites was five times the rate for blacks in 1995, and female children in Bangladesh were less likely to be brought to clinic than their male counterparts [2]. Examples demonstrating inequalities between various economic, ethnic, gender-based, and other social groups are replicated time and time again in all countries [2,3].

Of 192 countries, 39 have a health information system sufficient to support basic analysis of socioeconomic inequalities and health. Such a health information system would include the vital registration system coupled with a major household survey. Ninety countries have only a census, an old household survey, or no data at all, whereas the remaining countries fall into a “middling” category (a census and a recent household survey, or two household surveys) (L. Bambas, P. Braveman, N. Dachs, I. Delgado, E. Gakidou, et al., unpublished data).

The two issues of health inequalities and human rights overlap in a number of ways that enrich a perspective on health as a human right. A human rights framework can inform a minimally acceptable level of data collection in health information systems—that is, a core set of equity indicators—as well as the conditions surrounding the release and use of that data. In contrast to individual measures of health inequalities, populations as units of analysis are especially useful for examining achievement of human rights (as well as identifying health inequities) since they indicate patterns of differential opportunity in society among various social strata. A human rights-oriented indicator of health inequalities, then, would be composed of two measures: (1) A health measure, including health status, health care, and other determinants of health or the social/ economic consequences of ill health; and (2) A measure of social position/advantage (also called an equity stratifier or social stratifier) that defines strata in a hierarchy, e.g., income or economic assets, education, sex, or ethnic group.

Health differences across social strata in comparison with the most advantaged group or an absolute standard suggest inequities in health [4,5]. This interpretation of health inequities directly coincides with a human rights framework since it focuses on a broad concept of health as well as on means to and opportunities for health. Critical issues of methodology for using these indicators also need attention, including identification of appropriate measures of social position and how to meaningfully compare indicators across countries.

Consequently, the Equity Working Group recommended that health information systems should include the information necessary to create equity indicators, and that research into methodologies should be given due attention. On a practical level, equity strata and health indicators can be integrated into a number of existing data sources, including censuses, vital registries, household surveys, small areas data, and administrative data sources. Such empirical information not only clarifies distributions of health and achievement of rights but can also identify barriers to health and provide insights on multi-sectoral approaches in planning and interventions to support the most vulnerable populations.

## Accountability and Autonomy

When equity-sensitive information is collected, access to information is often restricted to the government and is rarely disaggregated to show differences between socioeconomic groups. But a human rights approach to health information implies not only particular content but also mechanisms to promote the effective use of information, including the public release of data in a useful form.

Confidentiality and privacy issues arise in relation to information disaggregated by equity stratifiers, especially in the context of the public release of such data. There are strong arguments for the public release of health equity information, including the fact that such information is a determinant of health, and that civil society can play a vital role in improving health opportunities, both directly and by influencing governmental priorities. The public's general lack of knowledge regarding patterns of health inequalities and their causes within societies further supports the need for the public release of such information.

If information is disaggregated for very small populations, such as within a village, particular individuals or households may be identifiable and feel their privacy rights were impinged upon. Therefore, the public release of equity-oriented information on health should be explicitly planned for in the development of health information systems. Principles to guide release of disaggregated data should be followed, and communities should have a voice in the decision to release highly disaggregated information when privacy rights might be compromised. The HMN Equity Working Group suggests development of international standards for collection and sharing of disaggregated data and its use. Given the potential conflict between the two interests, we should continue to investigate possible technologic and/or policy solutions.

## Mutual Responsibility

Another mechanism for promoting the effective use of information is to support cultures of equity-oriented decision-making. In addition to the public release of information, strategies should include supporting research on pathways of health inequities and interventions; building capacity for analyzing information and developing interventions; encouraging demand for equity-sensitive data in government and the public; and supporting broad participation in the promotion of health equity.

Standards for improvements and target dates for achieving a minimally acceptable information system should take into account differences in the resources and needs of those implementing changes. The Equity Working Group recommended that target dates be developed with countries to define and integrate core equity indicators into routine information sources. All countries should be able to achieve, within the next 5–15 years, at least the middle-level information system, which could be considered a minimum standard. However, the rationale for this standard is predicated on the assumption of significant financial and technical support being given to the effort, and on the least well-off countries receiving the most support (L. Bambas, P. Braveman, N. Dachs, I. Delgado, E. Gakidou, et al., unpublished data).

## Conclusion

These recommendations provide a strategy for strengthening decision- and policy-making by providing a stronger empirical base for human rights considerations. This equity-oriented empirical base could strengthen health rights, not only through health sector decision-making, but also through decision-making in sectors related to determinants.

The initial development stage of the HMN has now ended, and hopefully the refinement and implementation stages of the effort will begin within the next year. Regardless of the existence of a centralized effort, countries would greatly promote health rights by integrating equity issues into health information systems, releasing that information publicly, and supporting participation and decision-making attentive to the concerns of health equity and human rights.

## Abbreviation

HMN: Health Metrics Network
